# Supplementary material for: A lncRNA signature associated with tumor immune heterogeneity predicts distant metastasis in locoregionally advanced nasopharyngeal carcinoma
Source: Nat Commun. 2022 May 30;13:2996. doi: 10.1038/s41467-022-30709-6 (PMC9151760; doi:10.1038/s41467-022-30709-6)
Supplement: Supplementary file 1 — Supplementary Information [file 41467_2022_30709_MOESM1_ESM.pdf]

Supplementary Information

A lncRNA signature associated with tumor immune heterogeneity predicts distant metastasis in locoregionally advanced nasopharyngeal carcinoma

Liang et al.,

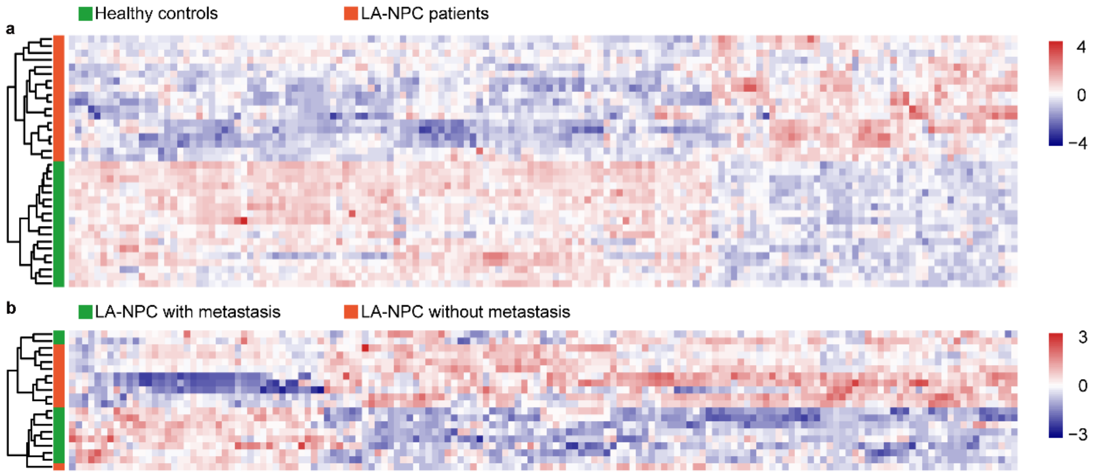

Supplementary Fig. 1. Hierarchical clustering of the differentially expressed lncRNAs. a Heatmap of expression profiling of the 149 differentially expressed lncRNAs between the matched 18 LA-NPC patients and 18 healthy controls. **b** Heatmap of expression profiling of the 149 differentially expressed lncRNAs between 10 paired LA-NPC patients developed with or without posttreatment distant metastasis. Both the row and column were unsupervised and clustered with the hierarchical clustering method. Source data are provided as a Source Data file.

Supplementary Table 1. Characteristics of 18 locoregionally advanced nasopharyngeal carcinoma (LA-NPC) patients and 18 healthy controls.

	LA-NPC<br>N (%)	Healthy controls<br>N (%)
Age (Mean ± SD)	45.1±9.0	45.3±8.6
Gender		
Male	11 (61.1)	11 (61.1)
Female	7 (38.9)	7 (38.9)

Supplementary Table 2. Clinicopathological characteristics of 10 pairs of LA-NPC patients developed with or without posttreatment distant metastasis.

	LA-NPC with distant metastasis N (%)	LA-NPC without distant metastasis N (%)
Age (Mean ± SD)	40.1±12.8	36.4±12.7
Gender		
Male	9 (90.0)	9 (90.0)
Female	1 (10.0)	1 (10.0)
T Stage		
T1	0 (0)	1 (10.0)
T2	0 (0)	0 (0)
T3	4 (40.0)	3 (30.0)
T4	6 (60.0)	6 (60.0)
N Stage		
N1	2 (20.0)	3 (30.0)
N2	5 (50.0)	5 (50.0)
N3	3 (30.0)	2 (20.0)
TNM Stage		
III	2 (20.0)	2 (20.0)
IV	8 (80.0)	8 (80.0)
Radiotherapy		
IMRT	10 (100.0)	10 (100.0)
Chemotherapy		
CCRT	5 (50.0)	5 (50.0)
CCRT+IC	5 (50.0)	5 (50.0)

Abbreviations: TNM, tumor-node-metastasis; IMRT, intensity-modulated radiation therapy; CCRT, concomitant chemoradiotherapy; IC, induction chemotherapy.

Supplementary Table 3. The number of events for different groups.

Number of events	Guangzhou training cohort (N=177)		Guangzhou internal validation cohort (N=177)		Guilin external validation cohort (N=150)	
	Low risk (n=126)	High risk (n=51)	Low risk (n=137)	High risk (n=40)	Low risk (n=118)	High risk (n=32)
Distant metastasis	10	20	11	17	14	16
Disease progression	28	22	23	21	38	19
Death	16	20	15	18	17	14

Supplementary Table 4. Multivariable Cox regression analysis of prognostic factors in the Guangzhou Training cohort of patients with LA-NPC

	Multivariate analysis		
Variable	HR	95%CI	*P* value
Distant metastasis-free survival			
lncRNA signature (high risk vs. low risk)	5.10	2.36–11.01	3.5e-05
N stage (N0-1 vs. N2-3)	2.41	1.04–5.56	0.039
EBV DNA (≥2000 vs. <2000 copies/ml)	2.86	1.15–7.08	0.023
Disease-free survival			
lncRNA signature (high risk vs. low risk)	1.88	1.06–3.33	0.030
N stage (N0-1 vs. N2-3)	2.82	1.49–5.33	0.001
EBV DNA (≥2000 vs. <2000 copies/ml)	2.10	1.10–3.98	0.024
Age (≥45 vs. <45)	1.58	0.91–2.76	0.107
Overall survival			
lncRNA signature (high risk vs. low risk)	3.09	1.57–6.06	0.001
N stage (N0-1 vs. N2-3)	2.46	1.16–5.22	0.018
EBV DNA (≥2000 vs. <2000 copies/ml)	2.95	1.28–6.80	0.011
Age (≥45 vs. <45)	2.19	1.12–4.28	0.022

We calculated hazard ratios and *P* values using two-sided Wald test in an adjusted multivariate Cox proportional hazards regression model, including variables that are significantly associated with clinical survival ($P < 0.05$) in univariate model using two-sided Wald test. These variables are all presented in the table above, no matter *P* value is significant or not in the multivariate model.

Abbreviations: LA-NPC: locoregionally advanced nasopharyngeal carcinoma; HR, hazard ratio; CI, confidence interval.

Supplementary Table 5. Multivariable Cox regression analysis of prognostic factors in the Guangzhou internal validation cohort of patients with LA-NPC

	Multivariate analysis		
Variable	HR	95%CI	*P* value
Distant metastasis-free survival			
lncRNA signature (high risk vs. low risk)	6.71	3.12–14.43	1.1e-06
N stage (N0-1 vs. N2-3)	2.47	1.04–5.87	0.041
EBV DNA (≥2000 vs. <2000 copies/ml)	1.84	0.71–4.75	0.211
Disease-free survival			
lncRNA signature (high risk vs. low risk)	4.13	2.27–7.50	3.3e-06
N stage (N0-1 vs. N2-3)	1.69	0.89–3.23	0.111
EBV DNA (≥2000 vs. <2000 copies/ml)	1.90	0.90–3.99	0.091
Sex (male vs. female)	1.85	0.82–4.21	0.140
Overall survival			
lncRNA signature (high risk vs. low risk)	5.44	2.72–10.87	1.6e-06
N stage (N0-1 vs. N2-3)	1.86	0.85–4.08	0.120
EBV DNA (≥2000 vs. <2000 copies/ml)	1.81	0.74–4.46	0.196
Sex (male vs. female)	2.50	0.86–7.22	0.091

We calculated hazard ratios and *P* values using two-sided Wald test in an adjusted multivariate Cox proportional hazards regression model, including variables that are significantly associated with clinical survival ($P < 0.05$) in the univariate model using two-sided Wald test. These variables are all presented in the table above, no matter *P* value is significant or not in the multivariate model.
Abbreviations: LA-NPC: locoregionally advanced nasopharyngeal carcinoma; HR, hazard ratio; CI, confidence interval.

Supplementary Table 6. Multivariable Cox regression analysis of prognostic factors in the Guilin external validation cohort of patients with LA-NPC

	Multivariate analysis		
Variable	HR	95%CI	*P* value
Distant metastasis-free survival			
lncRNA signature (high risk vs. low risk)	6.38	3.09–13.18	5.3e-07
N stage (N0-1 vs. N2-3)	2.87	1.23–6.72	0.015
Disease-free survival			
lncRNA signature (high risk vs. low risk)	2.74	1.58–4.76	3.5e-04
Overall survival			
lncRNA signature (high risk vs. low risk)	3.87	1.90–7.87	1.8e-04

We calculated hazard ratios and *P* values using two-sided Wald test in an adjusted multivariate Cox proportional hazards regression model, including variables that are significantly associated with clinical survival ($P < 0.05$) in the univariate model using two-sided Wald test. These variables are all presented in the table above, no matter *P* value is significant or not in the multivariate model.

Abbreviations: LA-NPC: locoregionally advanced nasopharyngeal carcinoma; HR, hazard ratio; CI, confidence interval.

Supplementary Table 7. Summary of the multivariable analysis of prognostic factors for distant metastasis-free survival and risk weight.

Variables	β Coefficient	HR	95%CI for HR	*P*	Weight
lncRNA signature					
Low risk	1	1			
High risk	1.64	5.14	2.38–11.10	3.13e-05	2
N stage					
N0-1	1	1			
N2-3	1.07	2.92	1.28–6.69	0.011	1

*We calculated the hazard ratios (HRs) and *P* values using two-sided Wald test in a multivariate Cox regression analysis, including lncRNA signature and N stage.

Supplementary Table 8. List of the real time RT-PCR primers used in this study.

	transcript ID	Gene ID	Selected in signature	forward primer (5'->3')	reverse primer (5'->3')
1	ENST00000561761	ENSG00000261220		CTGAGACCTCTTGCCTGACG	TGCAGTGAACACCTTGACCT
2	ENST00000430259	ENSG00000239415		ACTTGGCAACAGTCTTAGACCA	ACTGAAGAGTCTAAAATGAAGCAGG
3	ENST00000414890	ENSG00000223525		AAACGTGTGAAAATCGTATGAAATC	AGCTTGGCTTAAATAGATCAGAGGT
4	ENST00000573040	ENSG00000262038		CTGCTGTGGAGCCTTATGAAGT	CCAATATCTGGTCTCTCTTCCTTTC
5	ENST00000545709	ENSG00000250280		GAGCTTTCCTTGTTGAGGGGA	AAGCCACTGAGGGAGGGTTA
6	ENST00000605386	ENSG00000271614		TGAAGAGTGGCTGCAGATTC	CGATCAACTTTTCAGCAAATATCTC
7	ENST00000508640	ENSG00000248445		GCCATGGTTGGCTGACAGTA	TGTCGGCATCATCTTGTGGT
8	ENST00000499560	ENSG00000246982		TAACGGGCCTTTGCTGACTC	TTCCGTATTTTAGCAGCCACA
9	ENST00000605298	ENSG00000258940	√ lnc-TRAPPC6B-2	TTTCAGGACCAAAATGCGGC	TACTAAGCAATTTTGGCTACTCTG
10	ENST00000426737	ENSG00000230290		GTCTTCAGGCATCTTCTCTCCC	TCCTCTTTGTGGATTATCAGTGTC
11	ENST00000414750	ENSG00000234663		GGCCAGAAAACAACAGACTAACTC	AAGGCTTTAGAACTATTGTAGCCAA
12	ENST00000569274	ENSG00000260465		GGACCAAATGTGCAAACGGG	TTTGGGGCTGGTCTGGAATG
13	ENST00000450546	ENSG00000224857		TCTCCGGTGCATTCCATAGG	TGGCATTCAAAAGTATGATTCACAA
14	ENST00000505362	ENSG00000246763		GTGCGAGGCTCAGAAAGGA	GGCTCAGGAAATGAAACTGC
15	ENST00000510225	ENSG00000251171		TGGCTTGACAGGACTAGCAC	TGGACCAGTTCTCTGTTGCC
16	ENST00000421851	ENSG00000236535		CCTGATCTCAGGTGATCTACCTG	TGTTGACACTATCTACTTCAAGAT
17	ENST00000569353	ENSG00000261131		TTCAAACTAGGAATTGTATCCCCAT	AACTGTTTTGAGGAGCTTCTGTT
18	ENST00000449852	ENSG00000233871		AATCGCCTCAACTGCGGTAA	TCTTTTCCTCATCCGGCGAC
19	ENST00000510592	ENSG00000251555		GGGTCCATCTCTCTTGCCAT	AGAAACAGGTCCACTTGCCG
20	ENST00000587128	ENSG00000267058		AGCCCCTACGTGGTGAGTAG	TCTTCAGCGCCTCTCATCCT
21	ENST00000538219	ENSG00000237248		CGACATATTTTCCTGAAGGGTGT	GCCAAGTTATGCTCCGGCT
22	ENST00000462717	ENSG00000243410		GTCATCAGACTTCAATTTCACAGAC	ATTATATGCACAAAATGGAAGGCT
23	ENST00000512105	ENSG00000249621		GGGTTGGACAGGTAAGCCTC	TTGGCAGTGGGTACATGGTG
24	ENST00000484172	ENSG00000228775		CTTCATGCAAAGCCGAATTCCT	AGCAAGCAGTGAGCTCTCAATAA
25	ENST00000427722	ENSG00000232104		CTCACTACAGGATGGGCCG	ATGTGCTCATTTCGAGCCTCT
26	ENST00000444319	ENSG00000238121		AAGTCTTTGAAGGTGGGGAAGG	TTGGTTAGAACGTGTAGGGAGTG
27	ENST00000606285	ENSG00000272463		TCAGCACGCAAACACACAAC	AGGCAACTCTGGTGGCTTAT
28	ENST00000541775	ENSG00000205885		TTCGCCTGAGGTTACACGAC	GCCAGCATCTTCCTTTCCCT
29	ENST00000431803	ENSG00000230068		CAGACTTGGCATTTAGCTTGTCA	TTTTCAGGCGGGTAGCTGAA
30	ENST00000563434	ENSG00000259953		GCTGAATCACCACAGGCTTA	GGCTCAAGGGTGGACCAAG

31	ENST00000565519	ENSG00000261572		GGCCAGGATGAATCAGCGTA	GCCAGGAAAGCACAGCTCAT
32	ENST00000498714	ENSG00000242094		TCTTCAACTGCCTCCTTGGG	ATTCGGAAAGGGAAGCCGTT
33	ENST00000514452	ENSG00000249631	√ lnc-DRD5-10	GTCTTGATTGATAAGGGTGGCTG	CCCCATGAGGCAGTTAATGGT
34	ENST00000514446	ENSG00000250167		GAACTGACTCAAAGAGGCCCAG	AACCCAGGTGTTTGGCTGT
35	ENST00000596585	ENSG00000268996		CTGACTGTGGCATTTCTGCG	GCAGAGACCAGCAGAAACCT
36	ENST00000417120	ENSG00000228939		GCCCAGCTGTTGTTTTCAGTTT	GCTGCAGGCTCTAGAAAACAAAT
37	ENST00000565382	ENSG00000260018		CGCTCTAGGATCCCCGAAAA	CACGAGTCCATGACAGCAGG
38	ENST00000444482	ENSG00000228906		CACAAAACACGAGTTGGGGTC	ACCAACATTCACAAATTATTTTCCC
39	ENST00000431759	ENSG00000227533		AGGCTGCCGATCGGCT	AACTCCAGTCCAGCTTTCCA
40	ENST00000431813	ENSG00000236709		TCGGGAAACTCTGCTGTCAAC	GCTAAGGCTGTGGGCTTATG
41	ENST00000457043	ENSG00000231365		TAAAGGAGCTGAAATCTACCAGGG	CATCCCTTTCCTGCTCACTTCT
42	ENST00000565058	ENSG00000260711		TGTCTTCCCAGCAGAGGTGA	TGGAGGAATACACTTGGTGCC
43	ENST00000510682	ENSG00000248445		AGCCCAAATTGGATCACAGAAC	AAAGCTGGTTTCCTGCCCAA
44	ENST00000470435	ENSG00000244479		CCCACGGTGTCCTCGG	ACTGGAGGGGCACAGAGATA
45	ENST00000505677	ENSG00000246763		GTCCATCGACTCCAAGTCCTC	AAATCGCGGAAGGGGGTAGAG
46	ENST00000447430	ENSG00000233559		TTCTCCGTCACACCTCCTGA	GAGCATCCCAAAGGGGTTGA
47	ENST00000566282	ENSG00000259935		TCAGCCCCTAAGGCTCATAC	AACTGATTCACACAGCATCCCT
48	ENST00000457387	ENSG00000223745		AGGGTGTGGCCGTTTTACAT	GCCCAGGAGACCCAGTAAGT
49	ENST00000548096	ENSG00000257155		TTGGGTTTGTTGGCTCAGGA	ACAAACGTTTAGTAAAACGCTGC
50	ENST00000558443	ENSG00000259682		CACGTTGATGACATTTGCCGA	CCTGAAACACTTACCAACCAGC
51	ENST00000448834	ENSG00000234393		TGATGGAAGATGTTGGCCCC	CAAGGAGCCAAAAGGCTGTG
52	ENST00000501194	ENSG00000246792		TGCAGGAACTCTCACTGACAC	TCTCTGTGTGGCTATGGGTG
53	ENST00000570210	ENSG00000261114		GCCATACTGAGGCATGGAACA	CCTTTCAGGGCCATGCTAAC
54	ENST00000413828	ENSG00000232973		GGCGCCAGTTCTAAGCATT	CAGCAGGGGGTCTTGACTATTT
55	ENST00000564460	ENSG00000260316		TGCTCCTTGTGTGCAAACTG	CCTGGCCTTTGCTCGATTCT
56	ENST00000663557	ENSG00000232931		GAGGAACTCCTCCCCAACTC	TCAAGGGTATTGGCCTGAAG
57	ENST00000429681	ENSG00000235236		CATTGCACAGGCCAGTTCAATTC	TTCTTCTTCTTCTGTGCCCTCAA
58	ENST00000570612	ENSG00000226089		TGTTCATTTCATGGAAGGACGTT	AGTGTGCACTCAAATCTTTCTTTCT
59	ENST00000420195	ENSG00000224660		GTATTGACAATTCTGCCCTGTC	CTCTGATAGGCACGGTAGGT
60	ENST00000433377	ENSG00000235741		TTGGTTCTGAGGCACCCTCT	CTCCATCTGACAGCCCGTTC
61	ENST00000526206	ENSG00000254731		ATACTGCCCAACCAGCTCTC	TCTGCTTCTTGCCTCCAACA
62	ENST00000535078	ENSG00000205885		CCACCCTCTTGGAAATGCCT	CATCCTCCACTCGACCACAC
63	ENST00000531549	ENSG00000254556		GGAATTCCTGTCTCCCGCTT	CCTTGCTCTAGCTCCAGTCC

64	ENST00000561402	ENSG00000247809	√ NR2F2-AS1	CAGCACCGTTATTACTGAATTAGAT	TTCTTTATTTGCCTGGTGGAGA
65	ENST00000621103	ENSG00000233006		ATGCTGCTGCCCTAACAAGT	CACCTCCCTTGAGATGTTGCT
66	ENST00000567102	ENSG00000260296		AGGCTGCCTTCTAGTACCAAAT	AGGCAGAGTAATGCTTTTCCTGAT
67	ENST00000415982	ENSG00000235236		GCAGCACCACCAGAGACAAC	TTTTCTTCTTCTTCTGTGCCCGT
68	ENST00000565861	ENSG00000261270	√ lnc-CETP-1	AGCTGCCTTCCAGGGTAAAG	ACAAAACCACCCCCAGTTGA
69	ENST00000428176	ENSG00000233355		CTGACTGCCAGACTCCAAAGT	TCGGAAAAGTAGGAAAAGCAGAATG
70	ENST00000428667	ENSG00000233818		CCGAAGAGGATACGCACTGA	ATGCCAAGGTCATGTGGTGG
71	ENST00000517854	ENSG00000254271	√ lnc-CDK1-1	TTGAGGAGGGAGGAGAGTGT	AGACATGACAGCCACAGTCC
72	ENST00000538943	ENSG00000256020		GCAGCCTCTGGTCTTGTTGA	GTCAAGTTCGGCCTCCAGAA
73	ENST00000503685	ENSG00000251044		CCACCAGGCTCAATACCCAC	AAGGCACATGGAACTAGACTGG
74	ENST00000522897	ENSG00000249898		GGCAGACAGAGATAGAATTACAGAGC	TCACCATCCAGGAAGAATCA
75	ENST00000648961	ENSG00000285521		AGGCTTCAAACTCCCTCGACA	CCGCTGGTTCTCTTCCTCTATT
76	ENST00000592431	ENSG00000267475		CCAGCGACCGTTTGTCATTC	CATGGCAAGTCATATGCGCC
77	ENST00000436340	ENSG00000227338		GGCAGCAGTGAGAGTTTGTTG	CCTGGGAGCTTGTGGAAGAC
78	ENST00000509924	ENSG00000248846	√ LINC02065	AAGAGACAGGAACACAGGTTGA	AGGCTGACACCTGTTGGTGA
79	ENST00000424404	ENSG00000230316		CAAAGAGGCGCTACACTCCAAG	CAGTCTGGGCGAATCCAAAGT
80	ENST00000506059	ENSG00000248311		GCTGACCTGTGATGGAGTGT	GGAGTGTCCTTCTAGCCTGG
81	ENST00000579368	ENSG00000265962		CGGCTCCTGGATGCTTACAA	CGTTTTCGGTGAGCAAGCTG
82	ENST00000582324	ENSG00000233098		CCCCACTCTACTGAGGCTTG	TTCCCTCAGTCAGTATGTAGTCA
83	ENST00000655459	ENSG00000288046		TGTGCAGTCCACACAAGAGT	TCACTTTGCCACTAGATCTGCC
84	ENST00000505893	ENSG00000249695		GCATTTCGCCTTACTGGTGTG	CCTCAAGACCATGTGCTCCC
85	ENST00000438324	ENSG00000232756		GGCCATCTGACGCTGAGAAT	CTTTTTGGCTCCCCTCACCA
86	ENST00000570408	ENSG00000262343		CCAGAGGGAAGCAGAAACGA	CCTGGGGCCATTCTGATCTC
87	ENST00000568932	ENSG00000260658		ACAGTCCAAGGATGGTTGCT	GCATTCAGAGGTTCCCAAACT
88	ENST00000608442	ENSG00000272620		TTCTGCTGGGCACACTAAGG	GTGTCCTTCTGGCTCACCTC
89	ENST00000520598	ENSG00000253356		ATAACCCTTGTCTAGGAGCTGG	TTTGAGACAAGATGAGGGGGT
90	ENST00000520890	ENSG00000253408		TCAAGTGATTCTCCTGACTACAG	CACCGAAACTGTAGGGGCA
91	ENST00000427109	ENSG00000233901		GGGAGGATTCTGGGCATGTT	GTTGGGACAGCAAACGTAGC
92	ENST00000518988	ENSG00000253535		GGAAGGGAGGAAGAACCATC	TATTGGGAGAGAGGGCTCAG
93	ENST00000429588	ENSG00000230479		AGCGAAGGAATCTGGTGTGG	TGTCCTTCACCGTCCCATTG
94	ENST00000552324	ENSG00000257139		ACAAGCAACCAAGAACCACAA	GTTCGCAGCTATTAAGCCTGT
95	ENST00000422971	ENSG00000238120		AATGGAATGCAGCCACACCT	GGCCTCTATCTGACGGCTTC
96	ENST00000430281	ENSG00000234688		AGAGTCCTTGAAGCTCTACTGAGA	CCAAGCCAGAACCTGAACAT

97	ENST00000417932	ENSG00000226004		TCCCAAAGCCCACATGACTG	ACTTGACAGGGCTTTAGCGA
98	ENST00000447898	ENSG00000206195	√ lnc-POTEH-7	TTGTCTCAAGGAAATAGAGATTGTG	CCAACCATTCCTAGACAGTGTTC
99	ENST00000553157	ENSG00000228288		AGGGAGGGTAAATGGCCTTG	TTCCTGCTTCATGTGGTCAAC
100	ENST00000560800	ENSG00000247809		GCAGGTGATGGTCTTGTTTTCC	GATTTCTGCCCTTGTTATGCTT
101	ENST00000604724	ENSG00000270547		CATTCACAGCAGAGTTTCATCCA	GCATATCACTTTTATTTGTTGGCTC
102	ENST00000443554	ENSG00000229791		AGTCAACTACTGCTGAGGCAT	ACAGGATTGGTCGTTGAGTTGG
103	ENST00000358073	ENSG00000243155	√ lnc-STX6-2	TGGAGGTTCTGAAAAGAGGCA	TCACCCGCAATATTGTCCCT
104	ENST00000534431	ENSG00000255202	√ lnc-C11orf91-2	CAGGAGGAACCTGCAAAGGA	CACAGTCTGTCAGAGGCGAC
105	ENST00000446911	ENSG00000225953		CCTGCCTTAAGGTTGGAATCT	TTGGCTTGTTGCAGAGTACGA
106	ENST00000617777	ENSG00000215483		TCGATTGTGCTAGGGCTTGG	TTCCTCCCCTGAACCACTCA
107	ENST00000456688	ENSG00000231856		CCCCAAATCCTCATCAGCCTC	TAGAAGACTGTGTCCACAGAAGC
108	ENST00000603033	ENSG00000271409		ACTGCTCATGTGTCAGGCTC	TTTGCGGGGGAGAGAAAGTC
109	ENST00000626945	ENSG00000280780		TGCTCCAGCTCACGAATCC	GAGCACACCCGTCCTTTGA
110	ENST00000445708	ENSG00000224972		TAATTTGGGGGAAGACTGGTGG	CTTTTCCTGCAAACACTGTCCC
111	ENST00000549878	ENSG00000257284		CTAGCTGACAGTGACTTCGGG	AAGTCACACGGTGTTTGATGC
112	ENST00000443224	ENSG00000224215		CTTCCCCACAAGACTCCCAC	GTAGAGGAGGAAGCGAGCAG
113	ENST00000556035	ENSG00000230805		ATACATCCTCACGGTCCTCGT	TCTCAGCGAGGGGTTCACT
114	ENST00000429843	ENSG00000234293		AGCTCCCTCTGCTGTTTTCT	CAGCTCACTCTCAGGTGGAAG
115	ENST00000464242	ENSG00000244300		CCCTGGAGGTGGCTGTTTAG	ATCCCGGTAGGCACAGGTAG
116	ENST00000548051	ENSG00000231758		TGTGCACCTAACACTGCATCT	TTTTCACCACGCCCTGTAGT
117	ENST00000450451	ENSG00000237250		TCACCATCGGCAATAAGCCA	TCTGGGTCAGAGCACTGAAATC
118	ENST00000537068	ENSG00000255717		GGCTGAAGTTACAGGTGAGCA	AAGTGGAGTTATGGGAAG
119	ENST00000563722	ENSG00000260302		GAGGCAGCCAAAGTTAGCAC	AGAGCATAGCTTTTCACACCCA
120	ENST00000496829	ENSG00000243572		ATGAGTGGGGGTTCCTCTTCA	CAGGGCAGATGTTTGCACAGT
121	ENST00000528717	ENSG00000255337		GGAAGCCTTTGAAGACCCTGA	GGCATCATCTTGGCACTCAC
122	ENST00000429809	ENSG00000227143		AAATACAGCGGCATAAGG	TAGCGTGTTCATGTGAGATA
123	ENST00000520576	ENSG00000204949		GTCCCAGTGAAGATGATCCCC	GGGAGAACAGGACACCAACAG
124	ENST00000416909	ENSG00000223392		TAGGGGAGCTTGGAGGAACC	GGGGTTCTCACCTGCTCAAG
125	ENST00000513067	ENSG00000250604		AAAAGAGGTTTATTTGGCTCACT	TTGTTAGCCAGAGCAAGCTTC
126	ENST00000540625	ENSG00000255750		GACTCAGCCTCCTCAGACAAA	GAGCTGAGGCTTGAGCAATTC
127	ENST00000515487	ENSG00000248221		GTGGGGCAGGAGGAGGTAAT	AAACTGCTTGACGTGTGCTC
128	ENST00000553826	ENSG00000259163		CCTGAACCTTTCAGTGCAACA	TGCCAAACCTGCATGGAATC
129	ENST00000522228	ENSG00000253924		CCTGTTCTAGGACACCCCCA	CCAGTTTCCTCCGAGCCTTC

130	ENST00000436665	ENSG00000175772	GAGTGGATGAACATGTGCTTGG	TATCATCAGCTATGCGTGCG
131	ENST00000449017	ENSG00000244625	CAACTCACCACATGCCT	AGCAGAGTGACCTTTGGC
132	ENST00000565531	ENSG00000260237	GTTGTGAGTTTGGGAGACTTACTTCT	TGTGGCAAATTGAAATGGAA
133	ENST00000505567	ENSG00000250256	GAACAAAGCACCCCCTCTCT	TCCAGTCTTAACGGCGAACA
134	ENST00000435858	ENSG00000226089	TGACAGCTACCTTAATTCATCCAT	ACTTCTGTTTCCTTTAGTCATTTGT
135	ENST00000541404	ENSG00000256582	AGTTGGCCTCAGGAAGGAAC	GCCTCAGACCATCTGTCCTC
136	ENST00000506431	ENSG00000249601	TCAGAAGAGCCTCCCTCACA	CCAAGCCGCAGGAGTTTCTA
137	ENST00000652774	ENSG00000214719	AAAGAAAACTGAGCGAGCCG	TCATCGTATGCAGTGCTTGT
138	ENST00000559505	ENSG00000247809	AGAGTCGGAAGTGGGTGTCA	CCATCTTCTTCCATTGGCTATTCCT
139	ENST00000658801	ENSG00000230530	CGGTGGCCATCTTCATCATTG	AGAAACACACAGCACCCTCTG
140	ENST00000620874	ENSG00000227676	CTTCAGTGATGAGGTCTGGC	TCTTCTTAGGAATCAAGA
141	ENST00000561979	ENSG00000261765	CCACTTGGTGATTCCACACAC	AAGTAGTTGTTTTATTCACGGAAGC
142	ENST00000433475	ENSG00000228108	GCTTCTACAGCTCTGGCATGA	GCCACCACCAGCAAAACTTTA
143	ENST00000414085	ENSG00000236028	CAAACTCAGGACGTGGTGGA	AGGTACTGGGGCCTTCTCTT
144	ENST00000514011	ENSG00000248309	GACTTGGACGCTTTGAACCTC	AAGGAGAGCTGGCTGTGAAT
145	ENST00000504829	ENSG00000247311	AGCTTGAAGACCATGCCACA	TGCTGGGCAAGTTTTCCGAT
146	ENST00000423256	ENSG00000228403	CAAAGCTTCCAGACACAGAGC	AGGTAGGCAAACCTGCATGA
147	ENST00000419129	ENSG00000237477	AGTCACTGCCAGGGTGTTTAG	ACAGCATGTTGTTTGGTGCTT
148	ENST00000665554	ENSG00000274461	CTCTACAGCTCCATGAGACA	GGATAGTAAAGCAAGTGGG
149	ENST00000583521	ENSG00000263585	AAGGAGCCCTTTCCCCAAATG	GATTCCCCAACCTCAACCTCTC
150	ACTB	ACTB	GCATGGGTCAGAAGGATTCC	AGGATGCCTCTCTTGCTCTG
