# Supplementary material for: A lncRNA signature associated with tumor immune heterogeneity predicts distant metastasis in locoregionally advanced nasopharyngeal carcinoma
Source: Nat Commun. 2022 May 30;13:2996. doi: 10.1038/s41467-022-30709-6 (PMC9151760; doi:10.1038/s41467-022-30709-6)
Supplement: Supplementary file 3 — Description of Additional Supplementary Files [file 41467_2022_30709_MOESM3_ESM.pdf]

Description of Additional Supplementary Files

File Name: Supplementary Data 1
Description: Result of differential expression analysis of lncRNAs. The P values were determined using the two-tailed t-test. False discovery rate (FDR) represented adjustments for multiple comparisons
