# Supplementary material for: A lncRNA signature associated with tumor immune heterogeneity predicts distant metastasis in locoregionally advanced nasopharyngeal carcinoma
Source: Nat Commun. 2022 May 30;13:2996. doi: 10.1038/s41467-022-30709-6 (PMC9151760; doi:10.1038/s41467-022-30709-6)
Supplement: Supplementary file 5 — Reporting Summary [file 41467_2022_30709_MOESM5_ESM.pdf]

nature portfolio

Corresponding author(s): Na Liu, Ying-Qin Li, Jun Ma, Wei Jiang

Last updated by author(s): May 7, 2022

Reporting Summary

Nature Portfolio wishes to improve the reproducibility of the work that we publish. This form provides structure for consistency and transparency in reporting. For further information on Nature Portfolio policies, see our Editorial Policies and the Editorial Policy Checklist.

Statistics

For all statistical analyses, confirm that the following items are present in the figure legend, table legend, main text, or Methods section.

n/a	Confirmed	
☐	☒	The exact sample size (n) for each experimental group/condition, given as a discrete number and unit of measurement
☐	☒	A statement on whether measurements were taken from distinct samples or whether the same sample was measured repeatedly
☐	☒	The statistical test(s) used AND whether they are one- or two-sided<br>*Only common tests should be described solely by name; describe more complex techniques in the Methods section.*
☐	☒	A description of all covariates tested
☐	☒	A description of any assumptions or corrections, such as tests of normality and adjustment for multiple comparisons
☐	☒	A full description of the statistical parameters including central tendency (e.g. means) or other basic estimates (e.g. regression coefficient) AND variation (e.g. standard deviation) or associated estimates of uncertainty (e.g. confidence intervals)
☐	☒	For null hypothesis testing, the test statistic (e.g. F, t, r) with confidence intervals, effect sizes, degrees of freedom and P value noted<br>*Give P values as exact values whenever suitable.*
☒	☐	For Bayesian analysis, information on the choice of priors and Markov chain Monte Carlo settings
☒	☐	For hierarchical and complex designs, identification of the appropriate level for tests and full reporting of outcomes
☒	☐	Estimates of effect sizes (e.g. Cohen's d, Pearson's r), indicating how they were calculated

Our web collection on statistics for biologists contains articles on many of the points above.

Software and code

Policy information about availability of computer code

Data collection	Agilent Feature Extraction software (version 11.0.1.1) was used to acquire raw data of microarray. qRT-PCR was performed using the Wafergen SmartChip Real-time PCR system (Takara Bio USA, Inc., San Jose, CA) and qPCR raw data were acquired by Wafergen qPCR analysis software (version 2.8.6.1).
Data analysis	Quantile normalization, quality control and differential expression analyses of microarray data were performed with the GeneSpring GX v12.1 software (Agilent Technologies). All statistical tests were performed in R software (version 4.0.3). The batch effect was removed by the "sva" package (version 3.38.0). The least absolute shrinkage and selection operator (LASSO) was performed by the "glmnet" package (version 4.1.1), receiver operating characteristic (ROC) curve analysis was performed with the "pROC" package (version 1.17.0.1), immune infiltration estimation was performed with the "MCPcounter" package (version 1.2.0), and gene set enrichment analysis (GSEA) was performed with "Clusterprofiler" package (version 3.18.1). Pathologic images were analysed using HALO software (Indica Labs, USA) using Multiplex IHC algorithm (version 3.1.4). Essential scripts for model development and validation in multiple cohorts are available on the Github website (https://github.com/YL-L26/lncRNA_signature_for_NPC).

For manuscripts utilizing custom algorithms or software that are central to the research but not yet described in published literature, software must be made available to editors and reviewers. We strongly encourage code deposition in a community repository (e.g. GitHub). See the Nature Portfolio guidelines for submitting code & software for further information.

Data

Policy information about availability of data

All manuscripts must include a data availability statement. This statement should provide the following information, where applicable:

- Accession codes, unique identifiers, or web links for publicly available datasets
- A description of any restrictions on data availability
- For clinical datasets or third party data, please ensure that the statement adheres to our policy

The publicly available hallmarks gene lists used in this study are available in the Molecular Signatures Database (MSigDB) database (http://www.gsea-msigdb.org/gsea/index.jsp), and the immune-related gene lists are available in the ImmPort website (https://www.immport.org/home). The microarray data used in this study have been deposited at Gene Expression Omnibus under accession code GSE180272 (https://www.ncbi.nlm.nih.gov/geo/query/acc.cgi?acc=GSE180272). The remaining data are available within the Article, Supplementary Information or Source Data file. Source data are provided with this paper.

Field-specific reporting

Please select the one below that is the best fit for your research. If you are not sure, read the appropriate sections before making your selection.

☒ Life sciences ☐ Behavioural & social sciences ☐ Ecological, evolutionary & environmental sciences

For a reference copy of the document with all sections, see nature.com/documents/nr-reporting-summary-flat.pdf

Life sciences study design

All studies must disclose on these points even when the disclosure is negative.

Sample size	Our study had a power of 80% to detect a hazard ratio (HR) for distant metastasis of 2·83 based on an assumed 5-year DMFS rate of 79% in the high-risk group and 92% in the low-risk group with a two-sided log-rank test at a significance level of 0·05. We anticipated that about 31 events were needed from 170 patients in the training cohort to develop a signature.
Data exclusions	In the training cohort, 28 lncRNAs were excluded from the biomarker selection as their expression, which was detected by qRT-PCR, was below detection in more than half of the samples.
Replication	All attempts at replications were successful. Number of independent experiments and replicates are in the Statistics and Reproducibility section and the Figure legends section.
Randomization	Samples obtained from LA-NPC patients from SYSUCC were randomly classified into the training cohort and internal validation cohort.
Blinding	Blinding was not relevant to the study as it was retrospective in nature. The investigators were not blinded to outcome assessment, because results used were obtained using objective quantitative methods.

Reporting for specific materials, systems and methods

We require information from authors about some types of materials, experimental systems and methods used in many studies. Here, indicate whether each material, system or method listed is relevant to your study. If you are not sure if a list item applies to your research, read the appropriate section before selecting a response.

Materials & experimental systems

n/a	Involved in the study	
☐	☒	Antibodies
☒	☐	Eukaryotic cell lines
☒	☐	Palaeontology and archaeology
☒	☐	Animals and other organisms
☐	☒	Human research participants
☐	☒	Clinical data
☒	☐	Dual use research of concern

Methods

n/a	Involved in the study	
☒	☐	ChIP-seq
☒	☐	Flow cytometry
☒	☐	MRI-based neuroimaging

Antibodies

Antibodies used	List of antibodies used in this study:<br>anti-CD8 (1:800, #ab4055; Abcam)<br>anti-CD20 (HPA014341, 1:3000; Sigma Aldrich, Merck)
Validation	The validation of primary antibodies were described by their manufacturers in the following websites:

Validation	- anti-CD8 (1:800, #ab4055; Abcam) (https://www.abcam.cn/cd8-alpha-antibody-ab4055.html)<br>- anti-CD20 (1:3000, HPA014341; Sigma Aldrich, Merck) (https://www.sigmaaldrich.cn/CN/zh/product/sigma/hpa014341)

Human research participants

Policy information about studies involving human research participants

Population characteristics	We included 542 nonmetastatic LA-NPC patients and 18 healthy controls in this study. The clinical features of patients participated in the study were showed in Table 1 and Supplementary Table 1-2. Of the 504 patients in the training and validation cohorts, 246 patients < 45 years old and 258 patients > 45 years old, 368 males and 136 females, 67 patients in T1/2 and 437 patients in T3/4, 245 patients in N0/1 and 259 patients in N2/3, 148 patients with pretreatment plasma EBV DNA < 2000 copies/ml and 206 patients with pretreatment plasma EBV DNA ≥ 2000 copies/ml (except for Guilin external cohort), 88 patients with distant metastasis, 151 patients with relapse, 100 patients in dead status.
Recruitment	Retrospective study of 392 patients with locoregionally advanced nasopharyngeal carcinoma and 18 healthy controls at Sun Yat-sen University Cancer Center between July 2010 and December 2016 and 150 patients with locoregionally advanced nasopharyngeal carcinoma at the Affiliated Hospital of Guilin Medical College between September 2014 and October 2017. Patients received no compesation. The informed consent was obtained from all patients.
Ethics oversight	The institutional ethical review boards of the Sun Yat-sen University Cancer Center and the Affiliated Hospital of Guilin Medical College approved this retrospective study.

Note that full information on the approval of the study protocol must also be provided in the manuscript.

Clinical data

Policy information about clinical studies

All manuscripts should comply with the ICMJE guidelines for publication of clinical research and a completed CONSORT checklist must be included with all submissions.

Clinical trial registration	NA
Study protocol	This was a retrospective collection of tissue samples from patients with locoregionally advanced nasopharyngeal carcinoma.
Data collection	Retrospective study of 392 patients with locoregionally advanced nasopharyngeal carcinoma and 18 healthy controls at Sun Yat-sen University Cancer Center between July 2010 and December 2016 and 150 patients with locoregionally advanced nasopharyngeal carcinoma at the Affiliated Hospital of Guilin Medical College between September 2014 and October 2017.
Outcomes	The primary endpoint was distant metastasis-free survival (DMFS), and the secondary endpoints were disease-free survival (DFS) and overall survival (OS). We defined DMFS as the period from the first date of treatment to the date of first distant relapse; DFS as the period from the first date of treatment to the date of the first relapse at any site or death from any cause, whichever occurred first; and OS as the period from the first date of treatment to the date of death from any cause.
